# Multi-component gradient enhancement for accurate frost detection and quantification on leaf surfaces

**DOI:** 10.1038/s41598-025-09131-7

**Published:** 2025-10-06

**Authors:** Huan Song, Lijun Wang, Yongguang Hu, Jie Yang, Jinxing Niu

**Affiliations:** 1 School of Mechanical Engineering, North China University of Water Resources and Electric Power, Zhengzhou, 450045 China; 2 School of Agricultural Engineering, Jiangsu University, Zhenjiang, 212013 China

**Keywords:** Frost crystals, Background, Gradient enhancement, MCGE-frost, Surface percentage, Mechanical engineering, Natural hazards, Environmental impact

## Abstract

Accurate frost detection on leaf surfaces is critical for agricultural monitoring, yet existing methods struggle with segmentation errors caused by complex backgrounds (blurred, soil, weeds) and subtle frost-leaf texture differences. To address this, we propose MCGE-Frost, a multi-component gradient enhancement method that integrates color space analysis with gradient fusion theory. The algorithm extracts gradient features from individual color channels (HSV, Lab), applies adaptive weighting to enhance frost-leaf boundary contrast, and employs morphological filtering to suppress background noise. Experiments on leaf images demonstrate that MCGE-Frost achieves a total algorithmic error segmentation rate of 3.29%, significantly outperforming ExG (8.63%), OTSU (8.98%), and HSV (11.98%). The method reduces computational complexity by 40% compared to deep learning-based approaches while maintaining robustness across diverse backgrounds. MCGE-Frost achieves 0.8 s/image processing on GPU-accelerated systems, balancing accuracy and efficiency for edge deployment. Additionally, it improves the intelligence of frost quantification with minor manual calibration. This advancement supports real-time frost monitoring in precision agriculture, providing actionable insights for frost protection and crop management.

## Introduction

Frost on the plant surface is caused by natural advection or inverse radiation and generally occurs during the period from late autumn to early spring^1,2^. Frost forms on plant surfaces through radiative cooling, which chills leaves below ambient air temperature, enabling direct vapor-to-ice deposition when humid air reaches the frost point^3,4^. Frost reduces plant temperature, and without timely prevention, it can cause cell damage, yield decline, and quality reduction. Therefore, with the continuous improvement of technical equipment for frost protection in tea and fruit orchards, there is an urgent need for a set of frost formation detection means to provide judgmental output for frost protection operations so as to achieve high efficiency and energy saving^5,6^.

Frost refers to the white crystals formed on the ground or objects when the air close to the ground is cooled below the frost point under the influence of ground radiation cooling^7^. As a kind of weather phenomenon, it belongs to the surface meteorological observation. At present, there is a lack of meteorological observation equipment for frost monitoring at home and abroad^8^, and there are also few technologies and instruments for frost detection of plants^9^. Most of them rely on manual frost observation, which is difficult to avoid problems such as strong subjectivity, low observation frequency and sparse distribution of stations, and the disadvantages of high investment and maintenance cost are becoming increasingly prominent. It seriously affects the benefit of automatic observation. In recent years, mechanized frost prevention technology and equipment have been gradually popularized and applied in agriculture, but its control is based on a single temperature or temperature inversion difference as the empirical judgment condition^10^. Due to the lack of real-time detection/monitoring of frost amount parameters, timely and accurate prevention and control of frost damage cannot be achieved, resulting in low automation and intelligence level in practical application^11^. Therefore, it is urgent to develop an effective detection method for frost formation, which is an important prerequisite for realizing accurate early warning and prevention of frost formation and providing judgment basis for defrost operation.

With the development of image processing and machine vision technologies^12–18^, the detection technology of frosting has entered the era of intelligence. Zhu et al.^19,20^ used machine vision technology to observe frost phenomena near the ground, and determined potential frost phenomena by designing a carrier of dew and frost phenomena suitable for image observation, a frost detection algorithm based on image change modeling of condensation contact surfaces, and then fitting the similarity by the proposed numerical model. Based on the surface condensation image set collected by the surface automated observation equipment, Zhou et al.^21^ studied the classification and recognition of surface condensation images such as dew, frost and rain using deep learning methods, including the self-construction and expansion of the surface condensation image set, the classification method using traditional convolutional neural network and the classification method using improved convolutional neural network with multi-way feature fusion to further improve the recognition accuracy of surface condensation images. In addition, the current new types of deep learning-based cases related to defrosting or predicting frost events for rails^22–24^, while exemplifying deep learning methods, also expose the need for urgent exploration of plants suffering from radiation cryogenic-type frosts.

Also based on image processing techniques, Niroomand et al.^25^ used a digital camera to take timed pictures of frost on test specimens, and studied the thickness, mass, density, and surface roughness of frost formation under natural conditions by calibrating the frost formation images with MATLAB software, and concluded that surface roughness is related to the density of frost, and as the roughness of the frost surface increases, the density of the frost layer decreases was concluded.

Frost phenomenon also widely exists in heat pump air conditioning, low temperature refrigeration and aerospace and other fields^26,27^. Previous studies on frost formation mainly focused on the frost layer itself, that is, the frost formation law was studied from the macroscopic phenomenological point of view, but the microscopic characteristics of the early frost formation and the dynamic characteristics of frost crystal growth were less understood. The formation mechanism of frost on the surface of the object varies due to the different external environment and surface characteristics, and the physical quantities used for characterization or detection will also be different, such as: the critical appearance of frost crystals, the density distribution on the surface of the object or the size of the accumulation amount, these characteristics reflect the growth distribution characteristics of frost crystals/layers on the surface of the plant at macro and micro levels, and it is very important to distinguish and detect whether and how much frost occurs in the whole process of frost formation observation. As a result, microscopic imaging is superior in the characterization of the frost layer proper compared to a regular camera, however, the limited light source at night results in insufficient image clarity of images refracted on the frost layer in a large magnification field of view, thus low-magnification microscopy will be a key method to quantify the mid-range of frosting^28–31^.

This work focuses on the detection of sporadic to uniform distribution of frost crystals on the leaf surface during the frost formation period and the quantification of frost formation in this process. According to the actual field scenes, three different backgrounds of plant leaf frost image categories were collected and generalized, and a multi-component gradient enhancement (MCGE-Frost) model was proposed. The purpose of new method is to capture and detect the trend of condensation characteristics of frost crystal morphology on the surface of plant leaves under each common vegetation background, and to realize the accurate measurement of frost crystal morphology and frost amount during the evolution of frost on the leaf surface under complex background conditions.

## Material and methods

In order to calculate the percentage of frost area on the blade surface, the frost crystals and the leaves need to be accurately segmented. The natural background has a certain degree of influence on the segmentation results when acquiring the frost formation images of the leaf surface. Therefore, choosing the appropriate segmentation method for different natural backgrounds will significantly improve the accuracy of the surface frost area ratio. Due to the fact that frost crystals mainly diffuse and grow as dendrites on the surface of leaves during the middle stage of frost formation, we intend to use the characteristics of HSV images of frosted crop leaves and the proportion of frost area on the leaf surface as an indicator to characterize the size or state of frost during this stage.

Frosted crop leaves in the wild environment generally occur in a variety of complex scenarios, and the captured images will inevitably contain more non-target objects, such as stray leaves and soil clods. Some existing conventional segmentation algorithms are often applied to a single scene, and their generalization ability is limited and the actual effect is unknown for complex scenarios. Therefore, the current main image segmentation algorithms are used as the basis, and the model to be optimized is selected by comparing the segmentation effect of different methods, combining with the actual problems of the algorithms, to explore the image characteristics of frosty leaves under multiple environmental conditions, and finally construct an algorithm that can effectively segment frosty leaves. The algorithm that can effectively segment frosted leaves is finally constructed to realize the quantification of the proportion of frosted area on the surface of crop leaves in the mid-term process of frosting. We collected 120 leaf images (40 per background type: blurred, soil, weeds), with 30 images per frost level (Frost I: 0–30% coverage, Frost II: 30–60%, Frost III: 60–100%). Experiments were conducted under controlled laboratory conditions: temperature (–5°C to 0°C), humidity (70–85% RH), and 15-min observation intervals over 4 h to document frost evolution.

### Experimental devices and processes

**Fig. 1:**
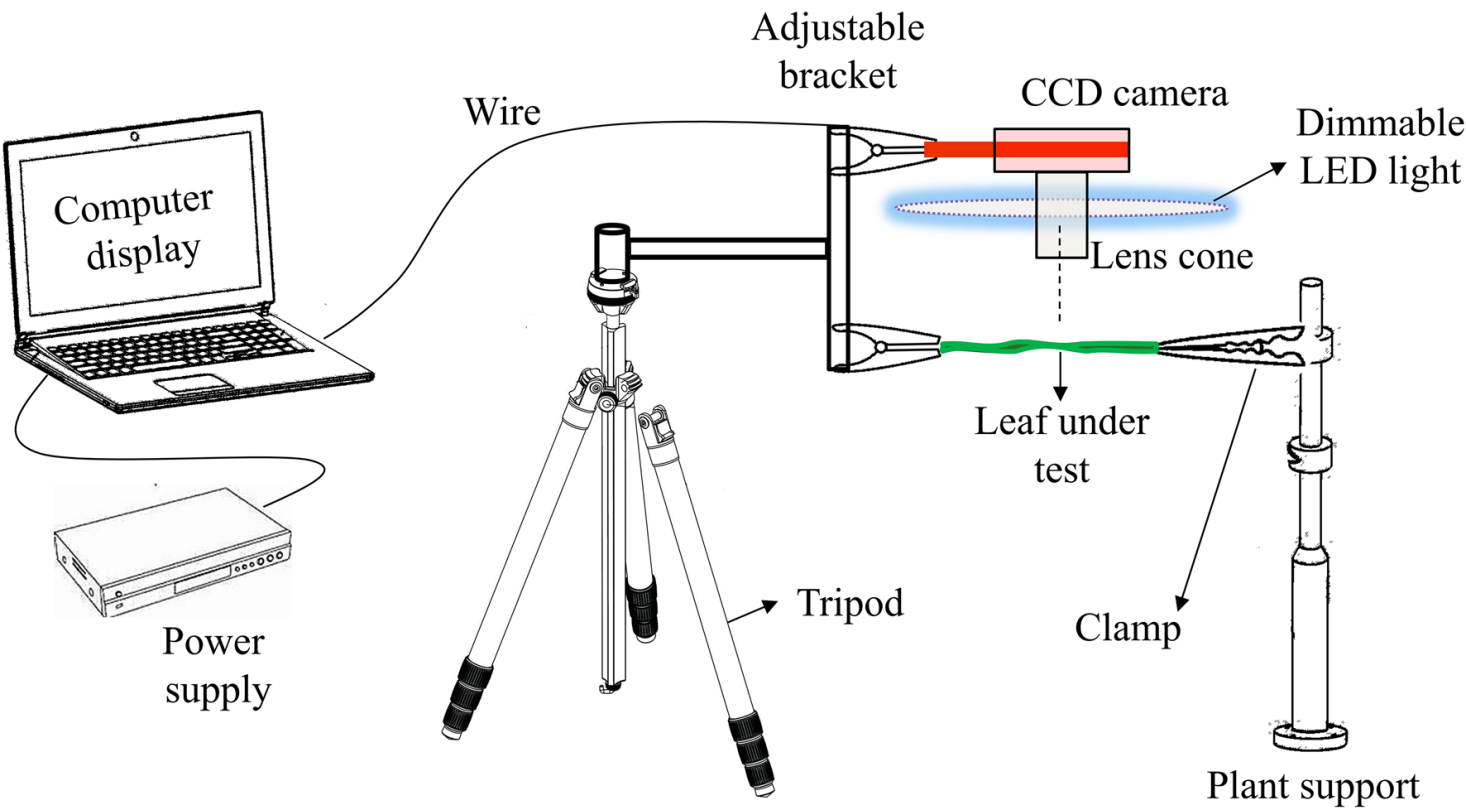
Structure and acquisition scenarios of an experimental device for leaf surface frost detection.

This research is dominated by portable imaging equipment and has been adapted to suit the data acquisition requirements of tea field scenarios. The structure of experimental device and a real scene of field acquisition are shown in Fig. 1. The device consists of a CCD camera, a dimmable LED light supplementation device and a computer display device, and the object to be measured is selected from the leaves of tea tree in winter. The imaging system contains a CCD camera, eyepiece, objective, and an adjustable bracket. The imaging system realizes the up, down, left and right all-round positioning and Angle control through the adjustable bracket, and the adjustable bracket is fixed with the tripod around the plant. In terms of power supply, AC 220 V voltage is supplied through wires to computer monitors, CCD cameras, and dimmable LED light.

Prior to imaging, leaf surfaces were gently cleaned with a dry soft cloth to remove debris, ensuring unobstructed frost observation. Image acquisition was performed under standardized lighting to minimize environmental variability. It avoids the exothermic effect on the leaf surface caused by scrubbing the surface with a damp cloth, and the temperature of leaf plummets to freezing point due to low temperature, thus affecting the natural observation effect. Then use plastic clamp to hold the sharp corner of the leaf, and insert the plant support into the ground to fix the plastic clamp, in order to make the surface of the leaf can be flat projection in the CCD camera. Then began to adjust the imaging system device, including adjustable stand placement and microscope viewing angle, while adjusting the light intensity and LED lamp position to eliminate overexposure and over-darkness as much as possible. Through a series of debugging operations, the final guarantee that the monitor can clearly project the whole image of frost on the leaf surface.

### Imagery multi-scale gradient enhancement (MCGE-Frost) model

#### Color enhancement

The HSV color model consists of three parts: Hue (*H*), Saturation (*S*) and Value (*V*), the three components in the HSV color model have higher independence compared with the RGB color model, and when the color of the plant leaves differs significantly from the surrounding environment, the RGB image of the leaves can be converted into an HSV image^32–34^.

The direct conversion of color images to greyscale images will result in the loss of some color features, making the detection results appear as edge disconnections or false connections. Therefore, the algorithm of this study chooses to perform gradient extraction on the HSV color space of the image.

The gradients were first extracted by Laplacian for each of the three channels of color components in HSV space, and then the final gradient image was formed by weighted fitting, the specific operation process is as follows:

Extracting the gradients of each channel component of *H*, *S* and *V*;

Calculate the weights of each component gradient image. The information entropy $I\left( {x,y} \right)$ of an image can be expressed by counting the probability of occurrence of all grey levels of the image:

1 $$I(x,y) = - \sum\limits_{0}^{255} {p_{(i,j)} \log p_{(i,j)} }$$

Thus, the image entropy values for the three-color components *H*, *S* and *V* are $I_{{\text{H}}} \left( {x,y} \right)$,$I_{{\text{S}}} \left( {x,y} \right)$ and $I_{{\text{V}}} \left( {x,y} \right)$ respectively;

Weighted image gradient, the final weighted synthetic gradient image is:

2 $$g(x,y) = \varepsilon_{{\text{H}}} g_{{\text{H}}} (x,y) + \varepsilon_{{\text{S}}} g_{{\text{S}}} (x,y) + \varepsilon_{{\text{V}}} g_{{\text{V}}} (x,y)$$

where $\varepsilon_{{\text{H}}}$, $\varepsilon_{{\text{S}}}$ and $\varepsilon_{{\text{V}}}$ are the weight of each gradient component, respectively.

#### Edge enhancement

For the segmentation of frosted leaf images in complex backgrounds, this paper uses the traditional method of processing the target image to show some of the details of the image, but there are many parts that cannot be displayed well, therefore, reducing the zero and low frequency components of the input image signal and extracting or enhancing the edges and details of grayscale images are key issues^35^.

Suppose the grey scale value of the coordinate’s pixel point is $f(x,y)$. For digital images the difference is used to approximate the derivative instead, bringing the obtained first-order gradient values into the above equation, the collation is obtained as follows:

3 $$h_{x} (x,y) = \sum\limits_{i = - 6}^{6} {f(x + i,y)t[i]}$$

where $t[i]$ is the coefficient by which the grey values of the neighborhood points are multiplied. The value of the gradient in the *y* direction is found and calculated as follows:

4 $$h_{y} (x,y) = \sum\limits_{i = - 6}^{6} {f(x,y + i)t[i]}$$

take the maximum of these as the gradient and use this to detect whether it is an edge point:

5 $$h(x,y) = \max [\left| {\left. {h_{x} (x,y)} \right|} \right.,\left| {\left. {h_{y} (x,y)} \right|} \right.]$$

#### Detection method of frost amount

Initially, frost appears as sparse white crystals (FCR ≈ 0%), gradually increasing to full coverage (FCR = 100%). With the frost crystals continue to grow until just covered the whole leaf, the process can be regarded as a period of leaf base color gradually become white dynamic time-varying process, when the leaf surface of the frost into the proportion of close to 100%.

Based on the characteristics of frost formation on the leaf surface, a definition suitable for detecting frost formation *δ* is proposed. The specific definitions are as follows:

Define the Frost Coverage Ratio (FCR) as the frost area of $A_{frost}$/the total leaf area $A_{leaf}$ at a single leaf scale, where the frost area $A_{leaf}$ is calculated and calibrated by means of continuous accumulation of image pixels.

The following formula is used to define the surface proportion of frost area:

6 $$Frost Coverage Ratio \left( {FCR} \right) = \frac{Frost Pixels}{{Total Leaf Pixels}} \times 100\% = \frac{{A_{frost} }}{{A_{leaf} }}$$

The segmentation principles of different background images are not consistent in the actual segmentation process, and there will be a lot of mis-segmentation in the direct extraction of leaf contour or frost crystal segmentation. For different backgrounds, there are two cases to calculate separately.

The total area of the leaves as follows in a blurred background:

7 $$A_{leaf} = A_{nf} + A_{frost}$$

here, $A_{nf}$ refers to the divided frost-free area pixels.

The total area of the leaves as follows in the image containing the soil and weed background:

8 $$A_{leaf} = A^{\prime}_{nf}$$

here, $A^{\prime}_{nf}$ refers to the frost-free area pixel after corrosion and expansion segmentation.

The key to image processing-based method for measuring the proportion of frost on leaf surface is to accurately segment the leaf and frost crystals. However, the evaluation of segmentation algorithm mentioned above is based on human eyes and has strong subjectivity. To objectively evaluate the segmentation accuracy of frost and leaf regions, we propose a mis-segmentation rate *R*_ms_ tailored for frost coverage quantification. This metric is calculated as:

9 $$R_{ms} = \frac{1}{n}\sum\limits_{i = 1}^{n} {\left( {\frac{{\left| {p_{s}^{(i)} - p_{a}^{(i)} } \right|}}{{p_{a}^{(i)} }}} \right)} \times 100\%$$

where $p_{s}^{(i)}$: number of pixels classified as frost/leaf by the algorithm in the *i*-th image. $p_{a}^{(i)}$: ground truth pixels manually annotated using Photoshop (reference standard). *n*: total number of test images.

A lower *R*_ms_ indicates better alignment between algorithmic outputs and ground truth. To ensure compatibility with broader segmentation literature, we additionally report Dice coefficient:

10 $$Dice = \frac{1}{n}\sum\limits_{i = 1}^{n} {\frac{{2 \cdot p_{s}^{(i)} \cap p_{a}^{(i)} }}{{p_{s}^{(i)} + p_{a}^{(i)} }}}$$

Among them, the smaller the value of *R*_ms_, the better the image segmentation effect using the algorithm. On the contrary, the larger the value of *R*_*ms*_, the worse the segmentation effect. As shown in Table 3,* R*_ms_ strongly correlates with Dice (*R*^2^ = 0.89), validating its consistency with established metrics while emphasizing proportional error sensitivity for agricultural applications.

11 $$G_{fused} = \sum\limits_{{c \in \left\{ {H,S,V} \right\}}} {w_{c} } \cdot \nabla I_{c} + \lambda \cdot \left( {G_{sobel} + G_{laplacian} } \right)$$

where *w*_*c*_ (channel weights) and *λ* (edge emphasis coefficient) are defined in Table 1.

**Table 1:** The parameter table.

Parameter	Description	Value/range
Hue weight (*w*_*H*_)	Weight for hue channel gradient	0.5
Saturation weight (*w*_*S*_)	Weight for saturation channel gradient	1.2
Value weight (*w*_*V*_)	Weight for value channel gradient	0.8
Edge emphasis (*λ*)	Balance between color and edge gradients	0.7
Morphological filtering	Threshold for noise removal	0.1–0.3
Kernel size	Size of morphological operation kernels	3 × 3, 5 × 5
Iteration count	Number of iterations for refinement	5–10

#### Model parameters and workflow

**Fig. 2:**
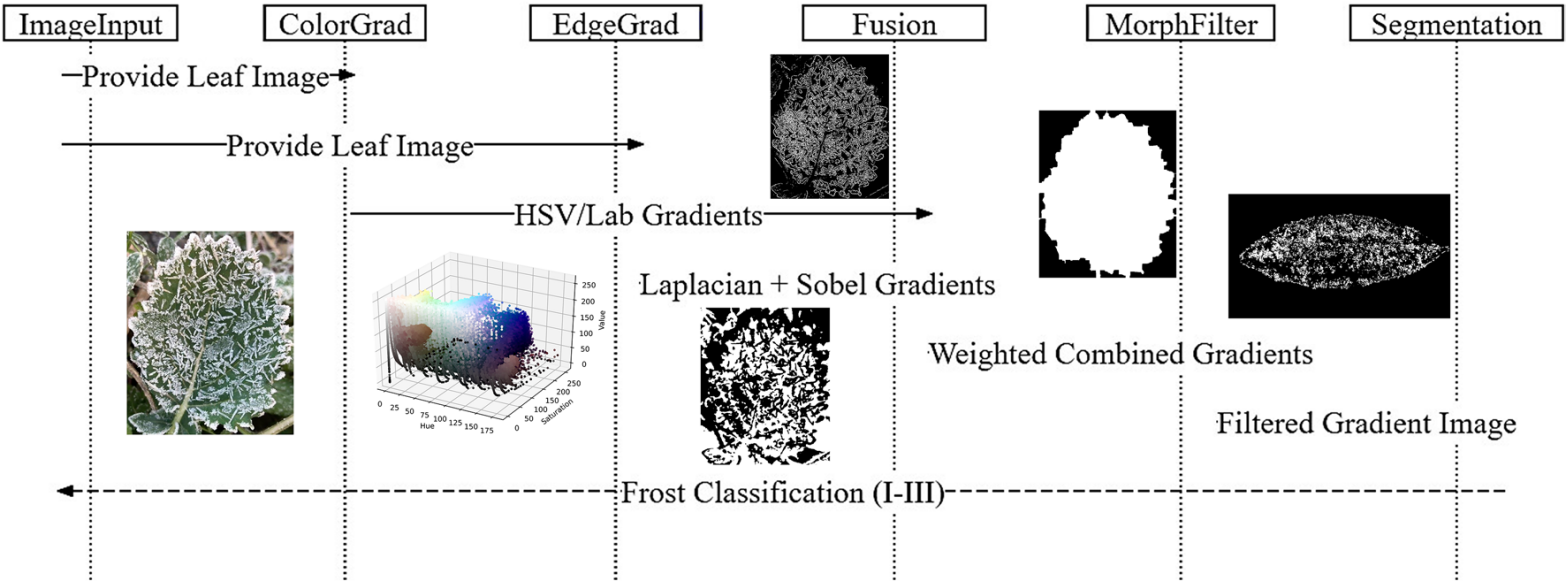
Workflow diagram of key steps with MCGE-Frost algorithm.

The proposed MCGE-Frost algorithm integrates color and edge gradient enhancement to address frost segmentation challenges (Fig. 2). Key steps include:

1. Input Preprocessing: RGB images are normalized and converted to HSV and Lab color spaces to enhance frost-leaf chromatic contrast.
2. Dual Gradient Extraction:
  Color Gradients: Gradient maps are computed for individual HSV and Lab channels (e.g., Saturation, Luminance).
  Edge Gradients: Laplacian and Sobel operators are applied to capture frost textures while suppressing leaf veins.
3. Weighted Fusion: Color and edge gradients are adaptively fused using channel-specific weights (Table 1), emphasizing frost-specific features under varying backgrounds.
4. Adaptive Morphological Filtering: Dynamic kernels (3 × 3 to 7 × 7) and thresholds (0.2–0.4) refine the fused gradient map, removing soil/weed noise while preserving frost structures.
5. Frost Segmentation: The processed map is thresholded to classify frost into three stages (Frost I: < 30%, Frost II: 30–60%, Frost III: > 60% coverage).
6. Post-Processing: Frost Coverage Ratio (FCR) is calculated and visualized for quantitative analysis.

Critical parameters (Table 1) were tuned to optimize performance across diverse field conditions.

### Feature extraction in different backgrounds

**Fig. 3:**
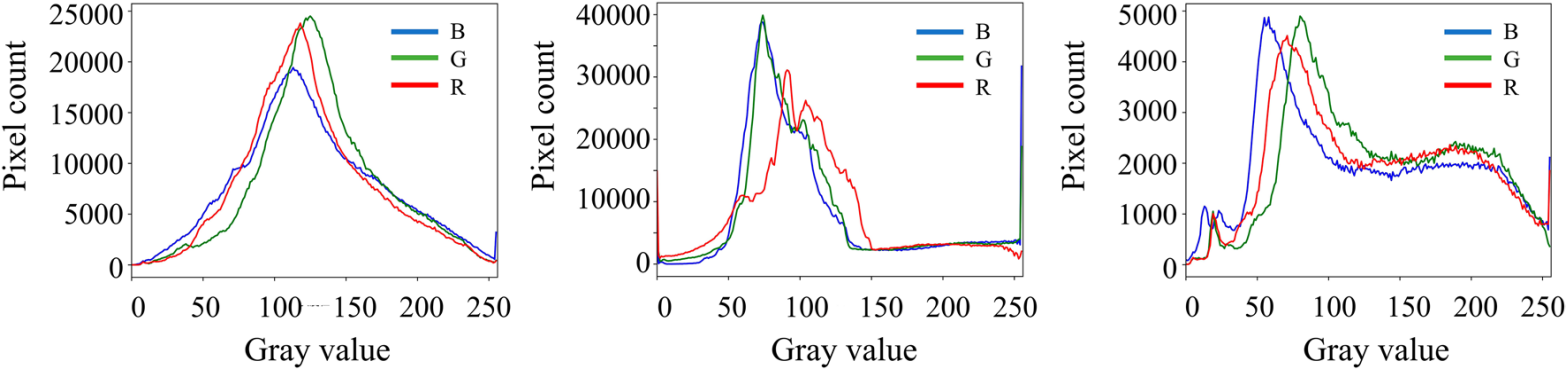
Color histogram of three grades of fresh tea leaves. (**a**) Soil background; (**b**) blurred background; (**c**) weeds background.

**Fig. 4:**
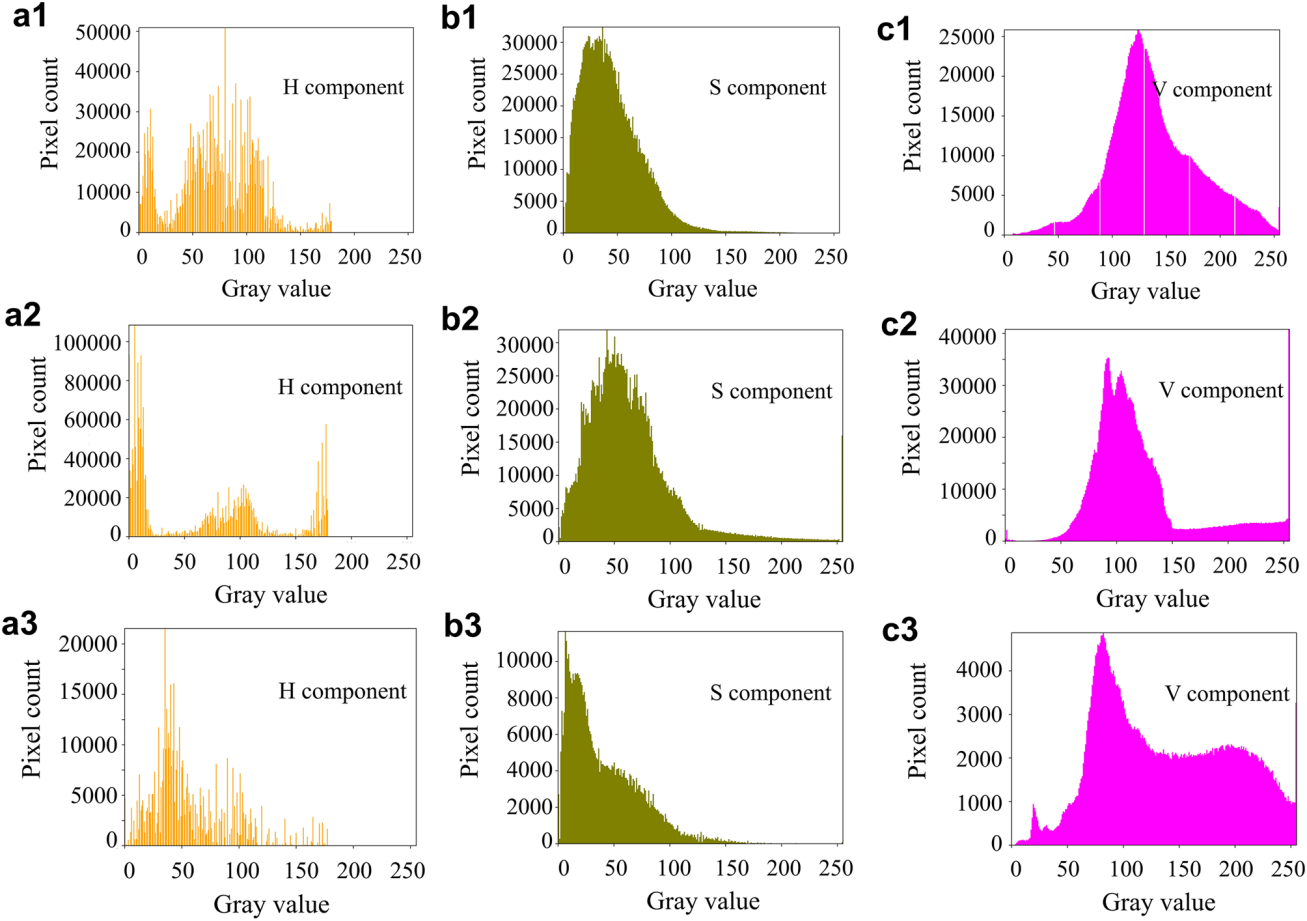
Histograms of each component of HSV for frost-forming images in three different backgrounds (soil, blurred and weed).

As a global feature, color is one of the more direct features in the image, and its distribution is generally described by histogram. In this paper, RGB and HSV color space are used together to differentiate the frost color feature of leaf surfaces under different background and frost conditions. After median filtering process, the color features were extracted from the images. The histograms in RGB space and the histogram pixel distribution in HSV space for the H, S and V channels for the frosted leaf surface images with three different backgrounds are shown in Figs. 3 and 4.

In Figs. 3 and 4, (a1), (b1) and (c1) are the histograms of the H, S and V components in the soil background, respectively; (a2), (b2) and (c2) are the histograms of the H, S and V components in the blurred background, respectively; (a3), (b3) and (c3) are the histograms of the H, S and V components in the weed background, respectively.

By counting the pixels of the color histogram of the frosted leaf surface image with multiple backgrounds, the pixels main distribution interval of frosted leaf surface image with soil background in RGB space is [50, 200]. The blurred background pixel distribution peaks in the 50–150 intensity range, and the frosted leaf surface image with miscellaneous leaf background is [50, 200]. The main distribution of pixels in the HSV space for the three grades of tea fresh leaf images is [50, 120] for the *H* channel, [0, 100] for the S channel, and [50, 200] for the *V* channel. From these three images, it can be seen that the green component occupies a larger component and is the key feature to segment the leaves and frost crystals.

### Color feature in light-medium-heavy frost

**Fig. 5:**
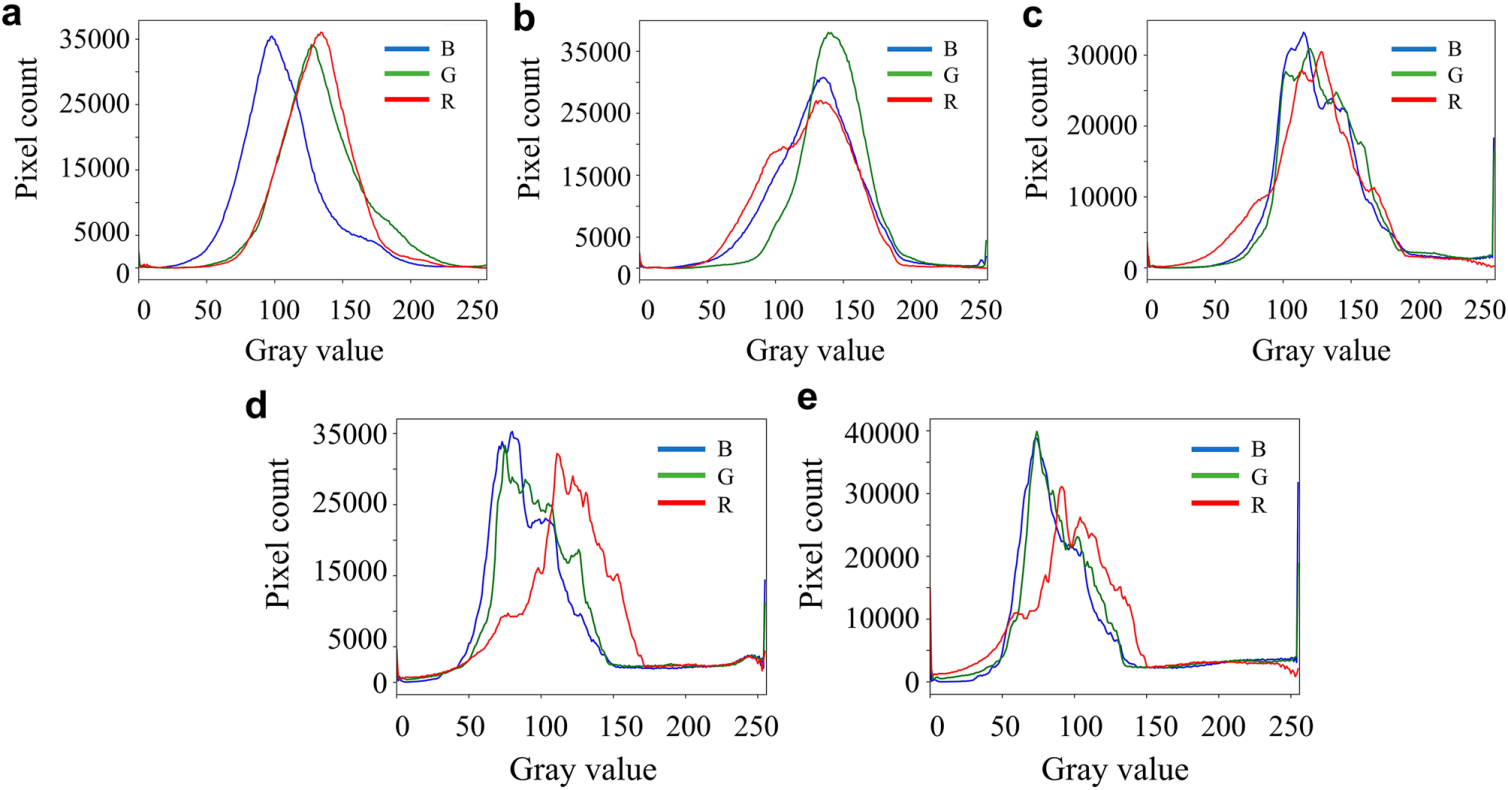
Color histograms of frost formation images on leaf surface at different frosting periods. (**a**) Non-frost leaf color light green; (**b**) Non-frost leaf color darkened; (**c**) Light frost leaf color dark green; (**d**) Frosty leaves dark green; (**e**) Frostier leaves dark green.

**Fig. 6:**
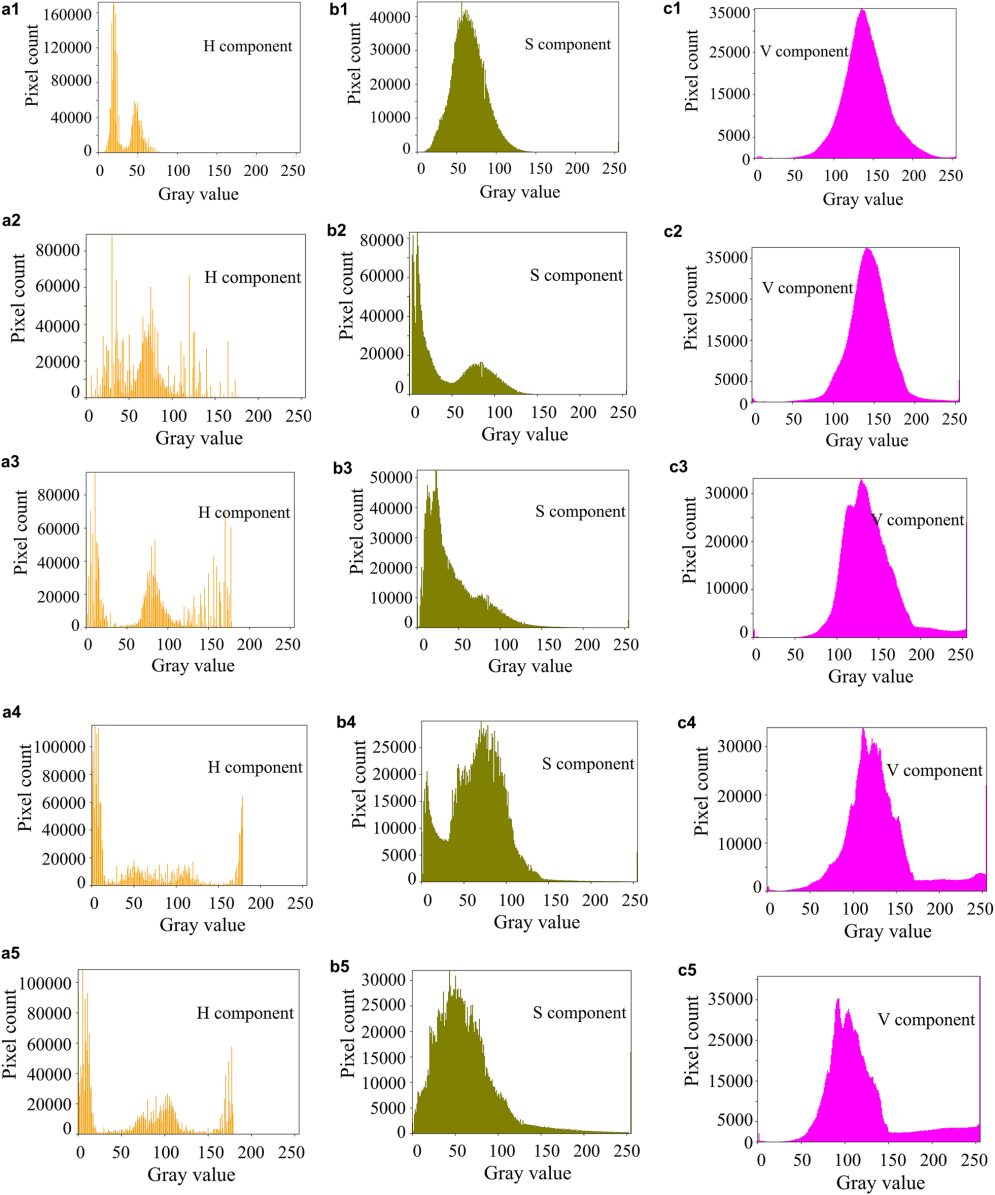
Histograms of each component of HSV for frost-forming images in the different frosting periods. (**a1**)–(**a5**), (**b1**)–(**b5**) and (**c1**)–(**c5**) are the histograms of the *H*, *S* and *V* components in period (**a**), (**b**), (**c**), (**d**), (**e**), respectively.

Figure 5 shows color histograms of frost formation images on leaf surface at different frosting periods. By counting the pixels of the color histogram of the frost image of the leaf surface in various frost states, it was found that in the RGB space, the main distribution range of the pixels of the frost image of the leaf surface in the frost-free night light green state was [50,200], and the *G* component was mainly around 130, and when the frost began to appear and increase in the process, the gray value of the *G* component was gradually low until it was around 70. During the actual observation in the field, it was found that the leaf color gradually changed from bright light green to dark green, which may be due to the slowing down of water transport inside the leaf due to the decrease of temperature, and the increase of water inside the leaf made the leaf color deepen. Figure 5 shows histograms of each component of HSV for frost-forming images in the different frosting periods. In HSV space, no frost leaf color light green state when the image of the *V* component shows a single-peaked normal distribution characteristics, the main distribution interval of pixels is [100,200], when the leaf surface performance for light frost and leaf color for dark green state, the distribution interval of *V* component has expanded to [150,255], indicating that the white pixels increase, and its brightness increased, reflecting the growth of frost crystal in the leaf surface coverage process. This can also be seen in the distribution of the H-channel histogram, where the number of pixels in the green component interval [35,77] rapidly decreases from the initial 60,000 to less than 20,000 (Fig. 6).

## Results and analysis

### Color and gradient feature fusion

According to the color features summarized and analyzed above, in order to clearly demonstrate the actual segmentation effect, the segmentation test was conducted on the leaf surface frosting images under the blurred, soil and weeds background, the percentage of leaf surface frost area was obtained in real time for different frosting states in the process of leaf surface frosting under the blurred background.

#### Blurred background

The three frost states are frost I, frost II and frost III, this paper defines frost I is the single leaf surface frost area ratio $0 < P_{lA} \le 30\%$ a; frost II is the single leaf surface frost area ratio $30 < P_{lA} \le 60\%$; frost III is the single leaf surface frost area ratio $60 < P_{lA} \le 100\%$; here $P_{lA}$ is the single leaf surface frost area ratio. The first is to use the HSV color space method on the blurred background of the leaf surface into a frost image, the specific segmentation process is shown in Fig. 5. (a), (b) and (c) are the raw image of frost level I, level II and level III state, respectively;

Following preprocessing, (a1), (a2) and (a3) are the raw image, HSV color space distribution, binarized segmentation image and color segmentation image of image (a), respectively; (b), (b1), (b2) and (b3) are the raw image, HSV color space distribution, binarized segmentation image and color segmentation image at the frost level II state, respectively; (c), (c1), (c2) and (c3) are the raw image, HSV color space distribution, binarized segmentation image and color segmentation image at the frost level III state, respectively.

**Fig. 7:**
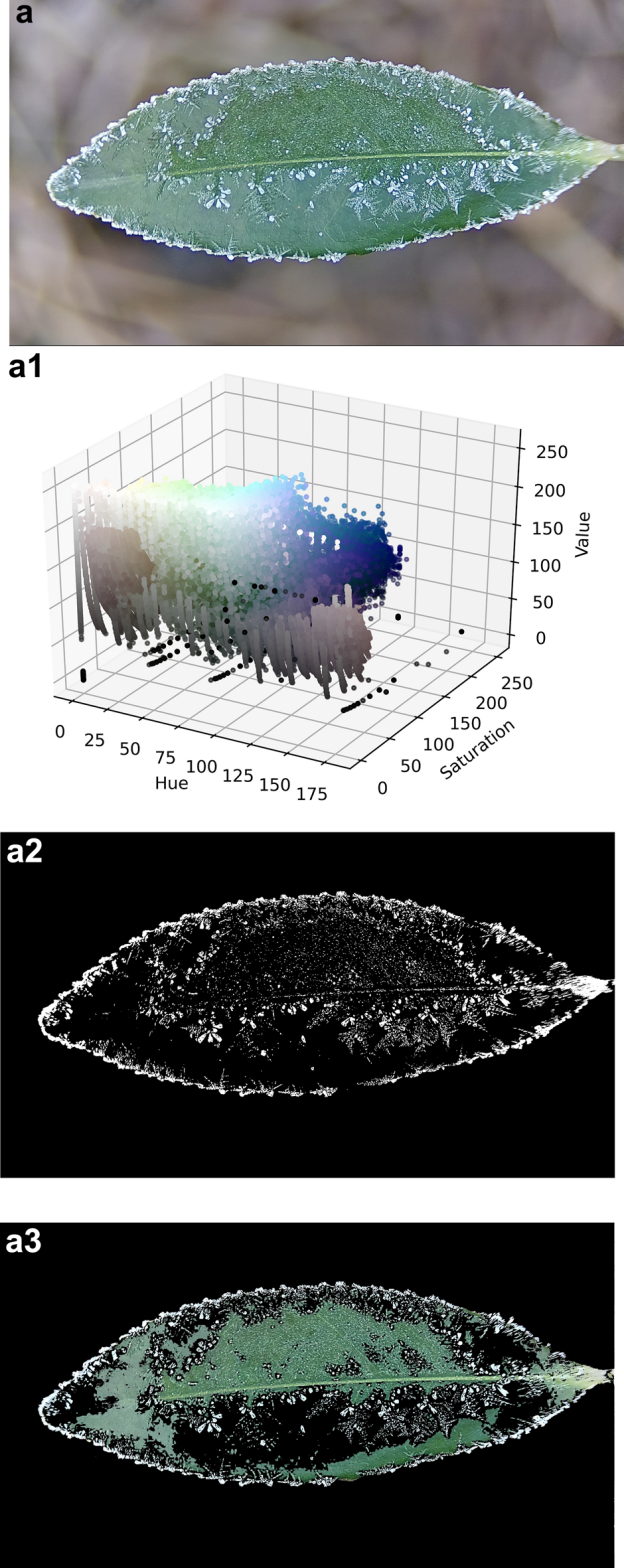

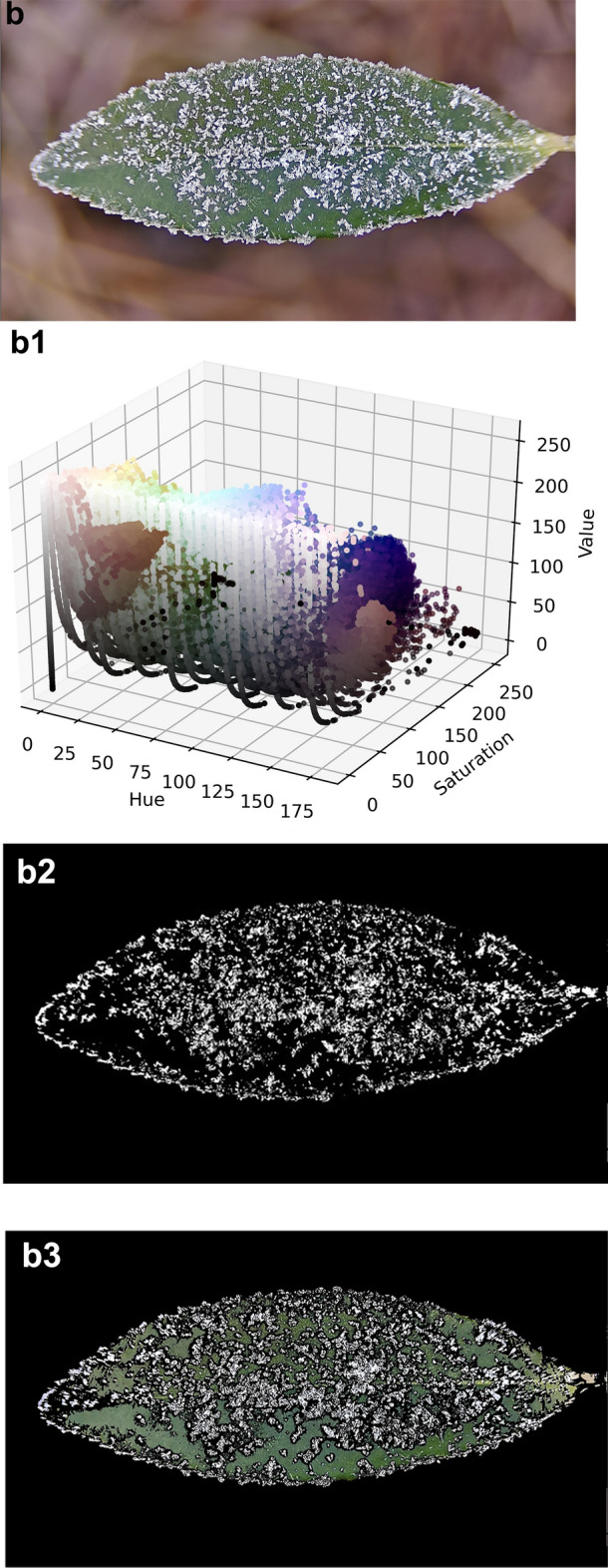

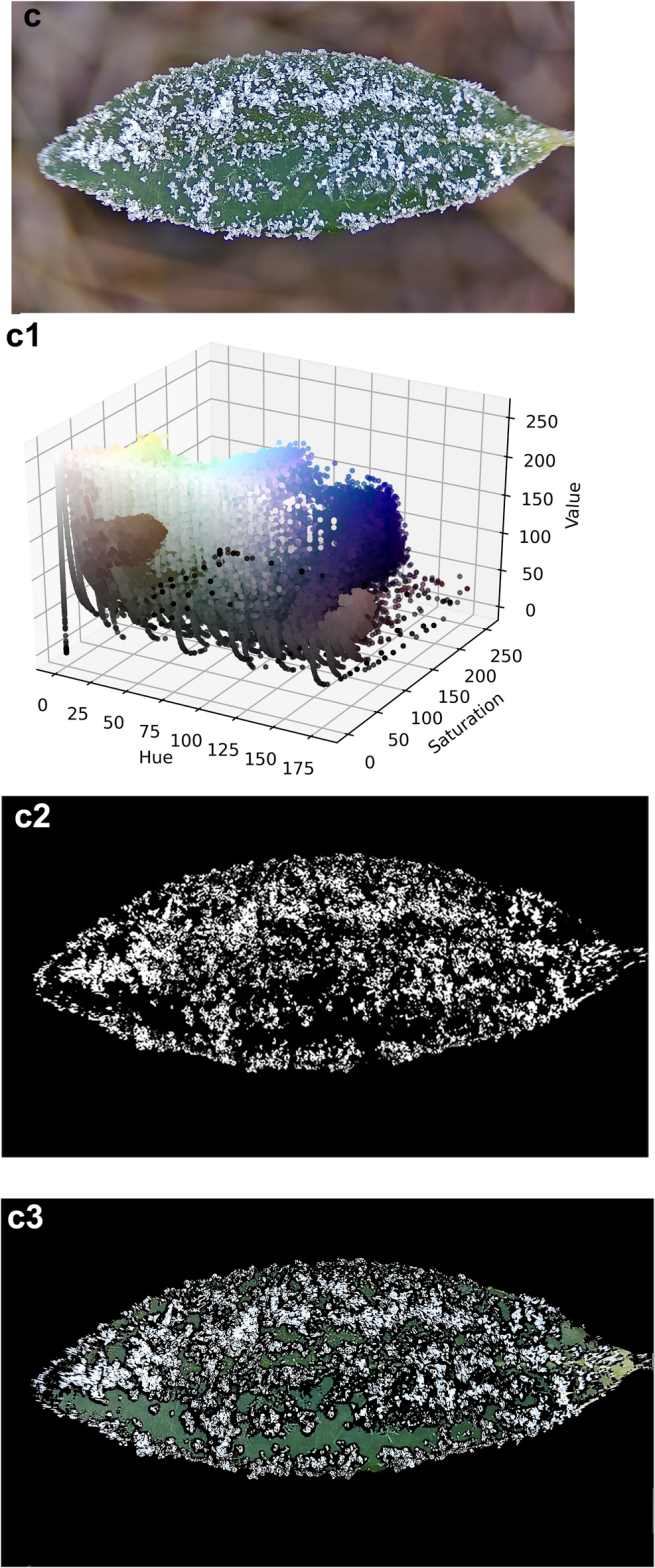
Frost crystal segmentation in different frosting states (frost level I, level II and level III state).

Figure 7 shows the segmentation of frost crystals on the leaf surface for different frost states. Frost crystals, leaves and the background are clearly and accurately distinguished, indicating that the advantage of the HSV color space segmentation-based algorithm is significantly higher than other algorithms. The RGB space color distribution is also examined here, and by comparing it with the HSV space color distribution, it is obvious that the frost crystal color and the leaf color tend to be more localized and visually separated in the HSV space. Since the green portion of the image spans almost the entire range of red, green and blue values, segmenting the frost-forming image of the leaf surface in RGB space based on the range of RGB values is not easy. The saturation and brightness of the frost crystals, the leaf and the background gradually differ as the percentage of frost area increases, which is a key point that can be used for segmentation. By looking at the HSV diagram, the color range of the target object is roughly determined, and the range of values here is 0 ~ 255. When selecting the range for the first time, try to choose a relatively large range so that the desired thing can be included in it.

#### Soil and weeds background

**Fig. 8:**
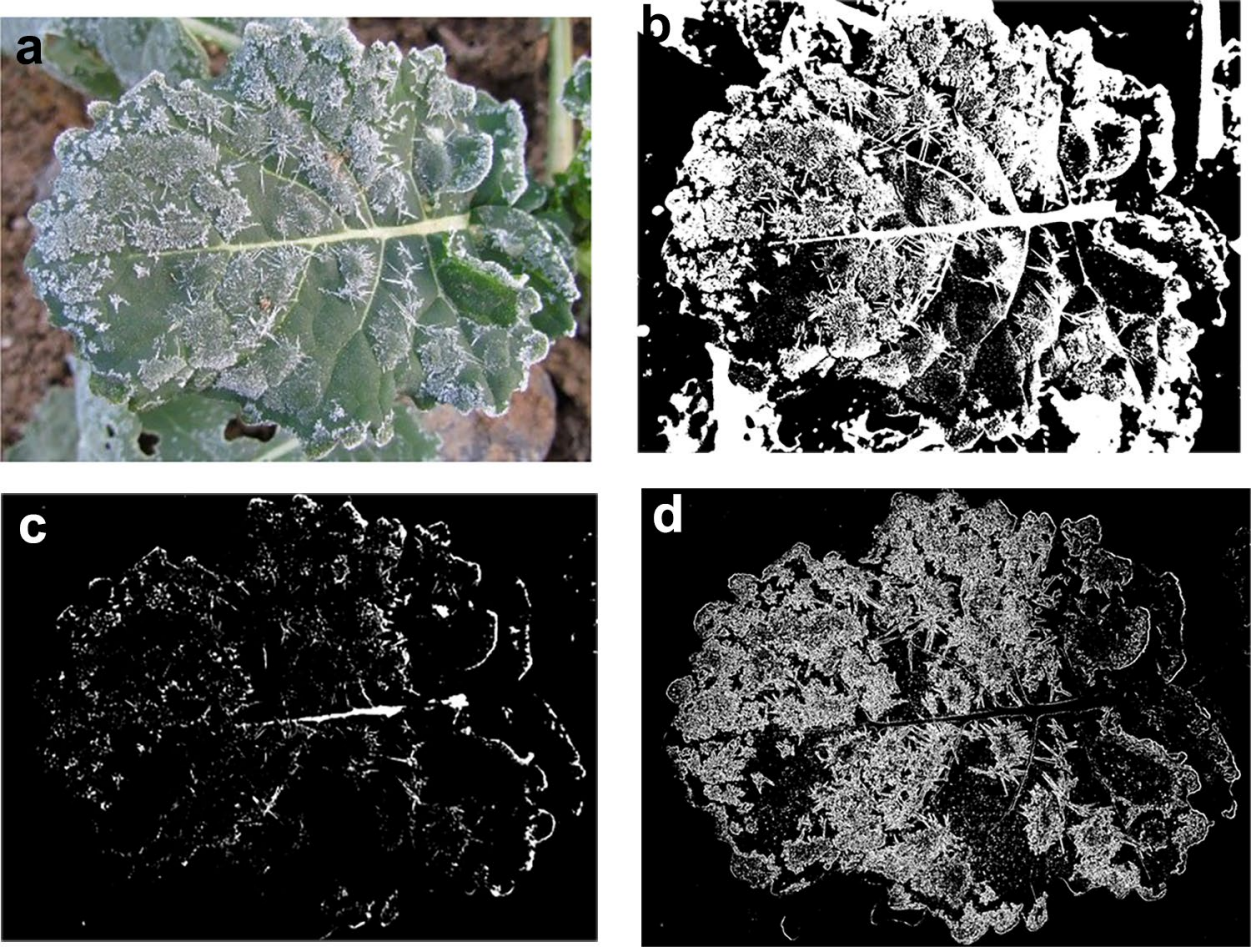
Segmentation results of different segmentation methods in soil background. (**a**) Original image *l*_2_; (**b**) segmented image *l*_2i_; (**c**) segmentation image *l*_2c_; (**d**) MCGE-Frost model segmented image *l*_2d_.

**Fig. 9:**
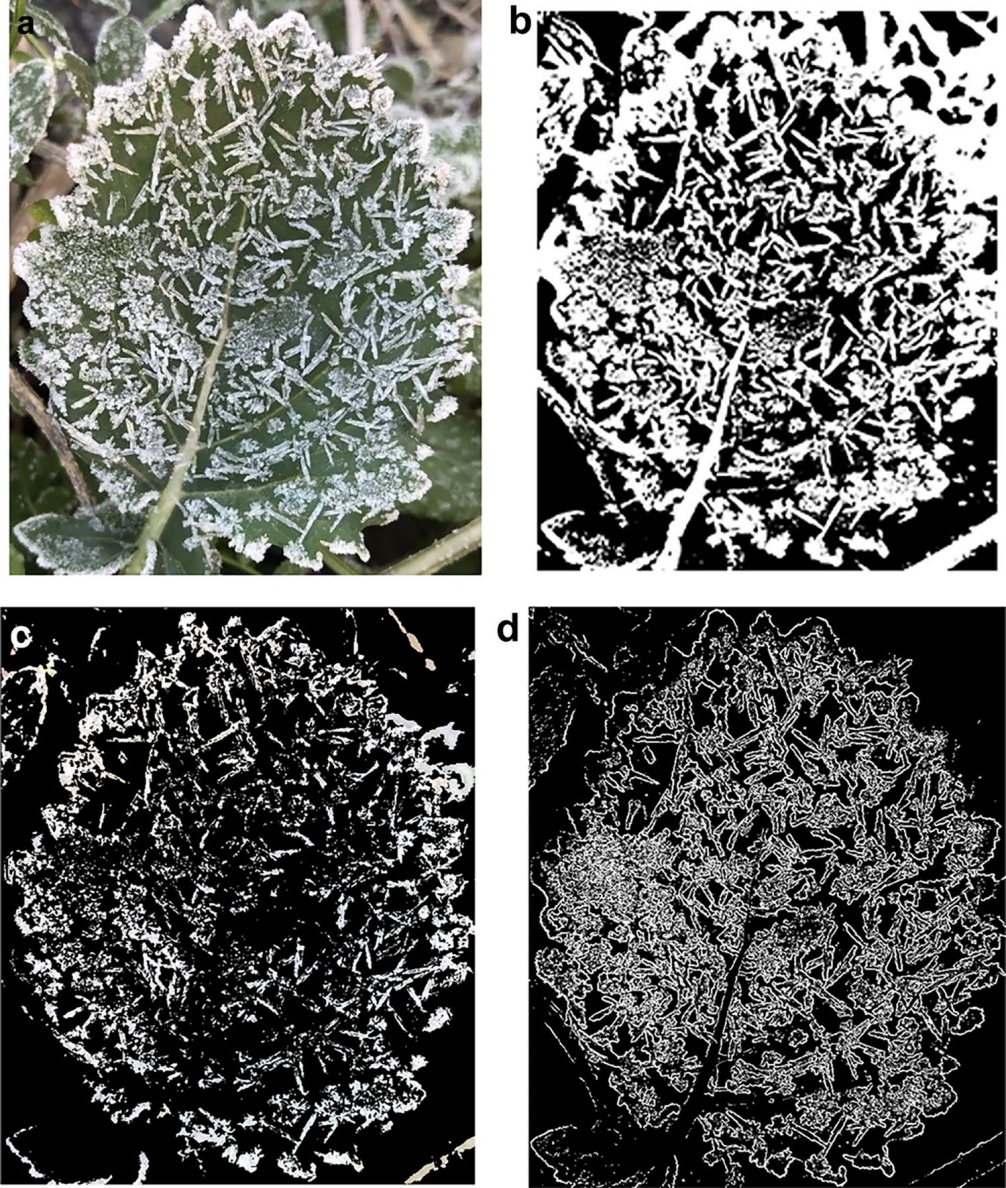
Segmentation results of different segmentation methods in weeds background. (**a**) original image *l*_3_; (**b**) segmented image *l*_3i_; (**c**) segmentation image *l*_3c_; (**d**) MCGE-Frost model segmented image *l*_3d_.

Traditional thresholding or color-space segmentation methods struggle to isolate frost regions in field images containing vegetation, soil, and weeds due to overlapping spectral features and background interference, particularly in distinguishing low-saturation frost from high-texture foliage. In order to eliminate the noise and reduce the segmentation error, the color scale segmentation algorithm based on gradient enhancement is constructed on the basis of HSV color space model. The results of segmentation are shown in Figs. 8 and 9. Where *l*_2i_ is the segmentation result using iterative method of thresholding, and *l*_2c_ is the segmentation result using color space method of thresholding, *l*_3i_ is the segmentation result using iterative method of thresholding, and *l*_3c_ is the segmentation result using color space method of thresholding.

From Figs. 8 and 9, it can be seen that the best result of the segmentation algorithm using the iterative method with multi-component gradient enhancement in this paper, such as Figs. 8d and 9d, the soil background has been completely and correctly segmented, and the real distribution of frost crystals on the leaf surface is also consistent with the original image, and the leaf edge contour is clear at the connection with the background. While only using the iterative method, such as Figs. 8b and 9b, the background both have serious over-segmentation problems, too much noise at the leaf edge, and the whole image cannot be clearly delineated. The overall effect of threshold segmentation using the color space method is slightly better than that using only the iterative method, the background noise is effectively reduced, especially in the soil background as in Fig. 8c, and in the weed background as in Fig. 9c, the edge of the leaf is obviously enhanced, but still need to further solve the problem of more background over-segmentation.

### Typical algorithm processing analysis

#### Extra-green

In order to contrast the actual segmentation effect of the algorithm sharply, three different backgrounds of frost-forming leaves are selected here, as shown in Fig. 9, which are frost-forming leaves *l*_1_ in the blurred background, frost-forming leaves *l*_2_ in the soil background, and frost-forming leaves *l*_3_ in the weed background. (a1), (b1) and (c1) are the color segmented images with blurred background, traditional image (soil and weeds) background, respectively; (a2), (b2) and (c2) are the binarization segmented images with blurred background, traditional image (soil and weeds) background, respectively.

**Fig. 10:**
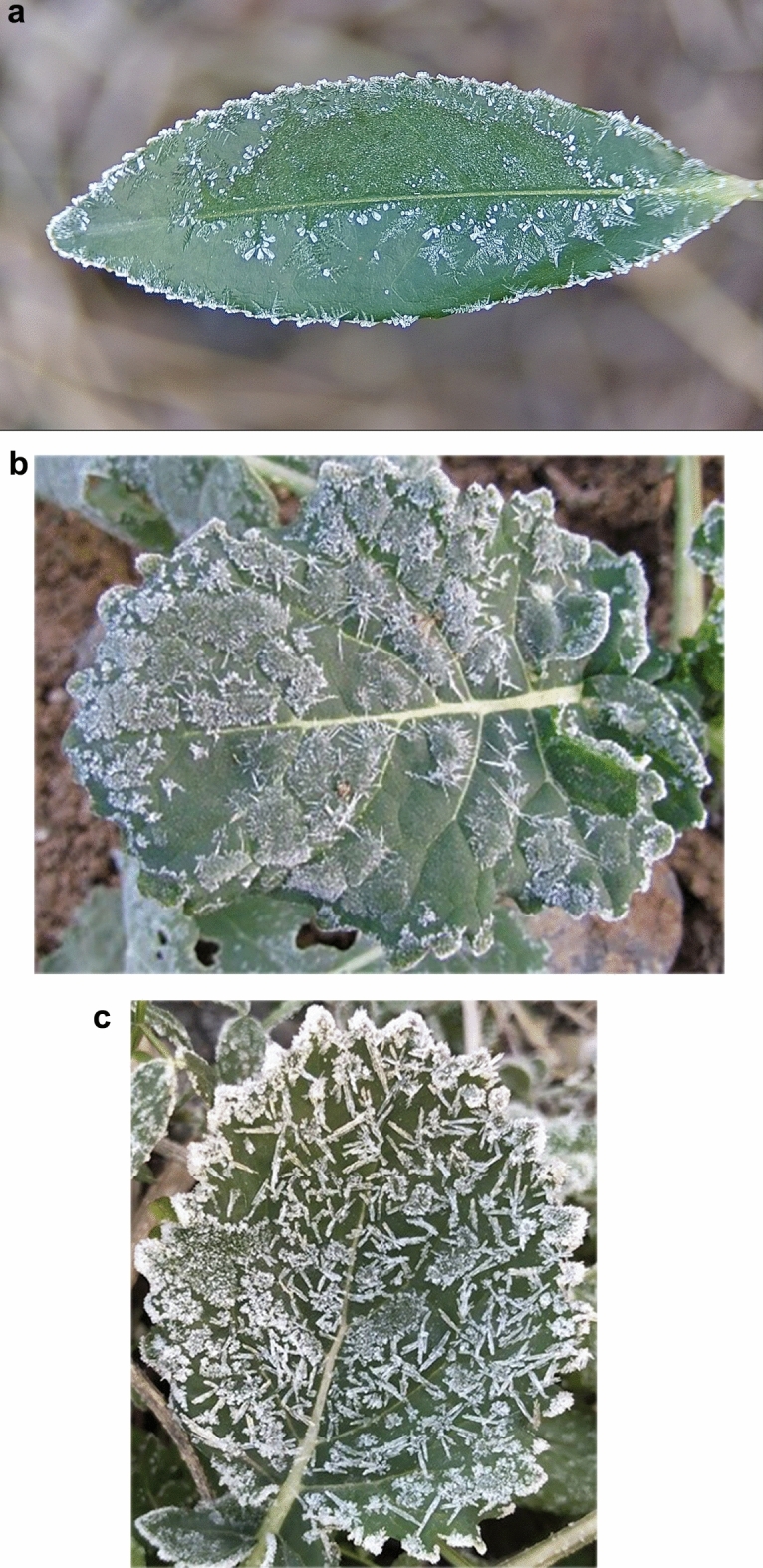
Images of frost-forming leaves before segmentation in three backgrounds. (**a**) Frost-forming leaf *l*_1_ in the blurred background; (**b**) frost-forming leaf *l*_2_ against the soil background; (**c**) frosted leaf *l*_3_ in the weed background.

**Fig. 11:**
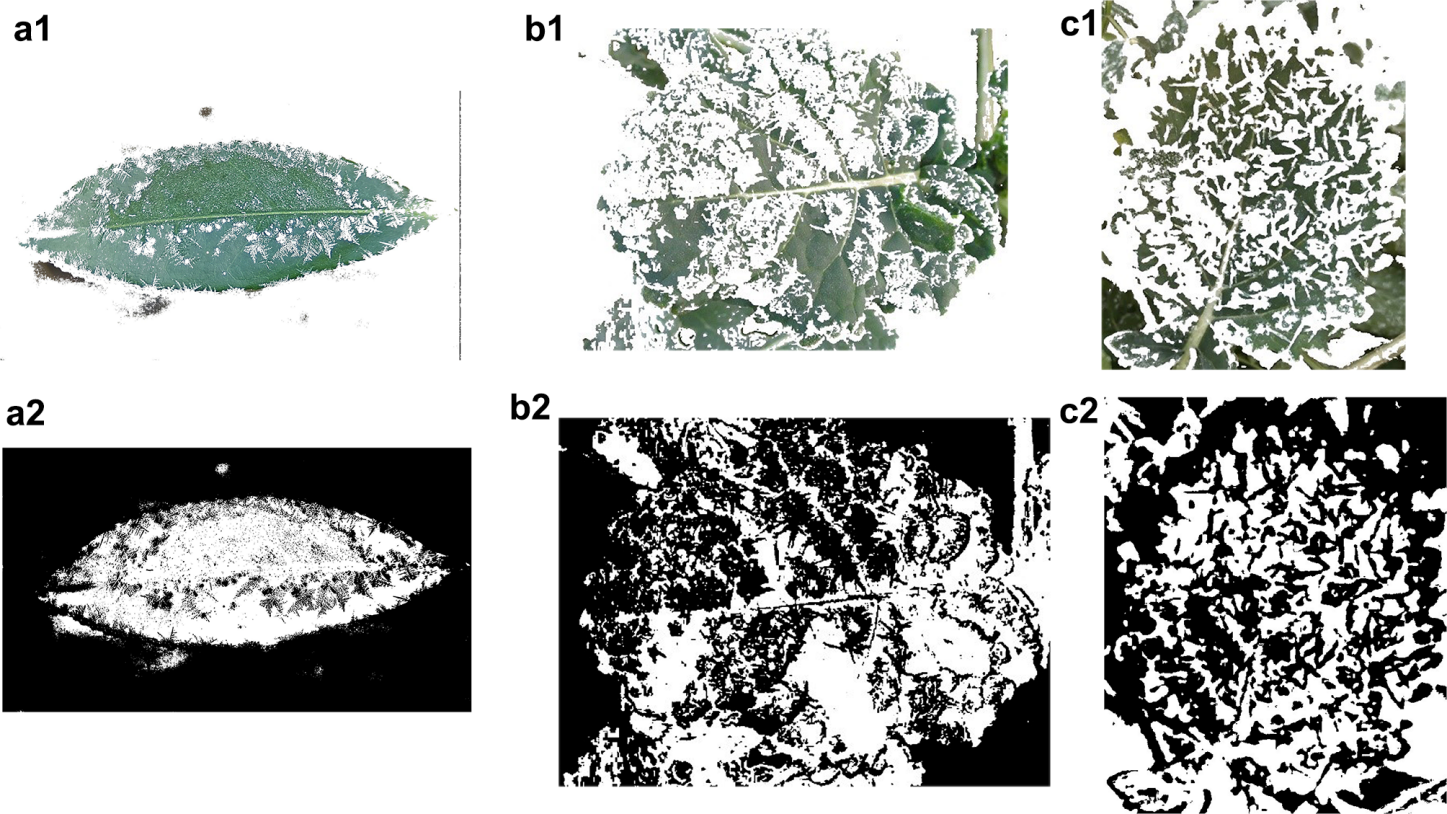
Images after segmentation with Exg algorithm in different backgrounds (blurred, soil and weed background).

From Figs. 10 and 11, it can be seen that the Exg algorithm segmentation for into frost leaf image of the overall segmentation effect is not good, especially in the traditional image (soil and weeds) background, in the image local easy to cause the image of the over-segmentation phenomenon, the background area identified as the target area, in the figure for the block of white areas, it can be seen that the Exg algorithm is not suitable for scenes with complex background, the frost leaf background requirements are high, if you want to achieve good segmentation effect is to eliminate the surrounding background noise.

#### K-means

**Fig. 12:**
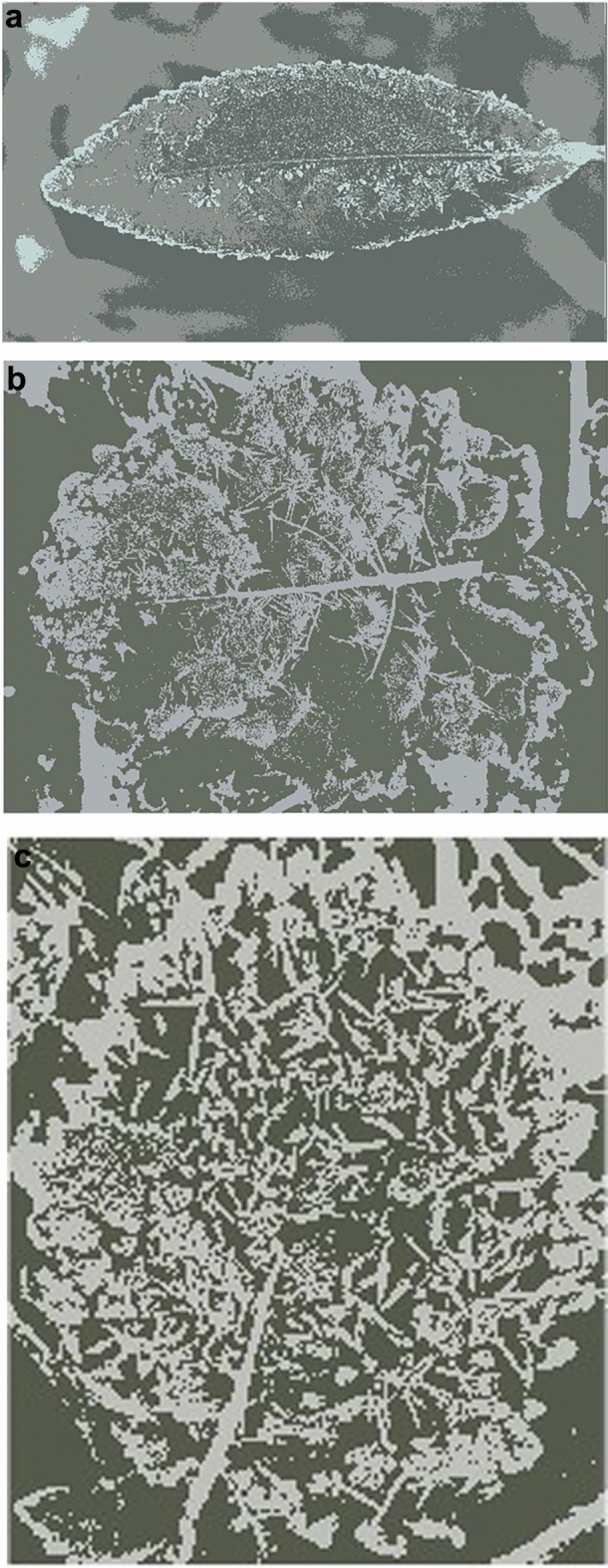
K-means segmented image after binarization process. (**a**) Segmentation results of frost-forming leaf *l*_1_; (**b**) segmentation results of frost-forming leaf *l*_2_; (**c**) Segmentation results of frost-forming leaf *l*_3_.

As shown in Fig. 12, the results of segmentation of the adult frost leaf image using K-means clustering algorithm. It can be seen from the comparison graphs before and after segmentation that the background area is over-segmented after segmentation, especially the edge part of the frost-forming leaf under the traditional background, which is not obviously distinguished from the background area, and the effective information of the surface frost crystals is lost, as shown in Fig. 12b,c, which will largely affect the subsequent results of feature extraction and frost area percentage of the frost-forming leaf image.

#### Otsu

**Fig. 13:**
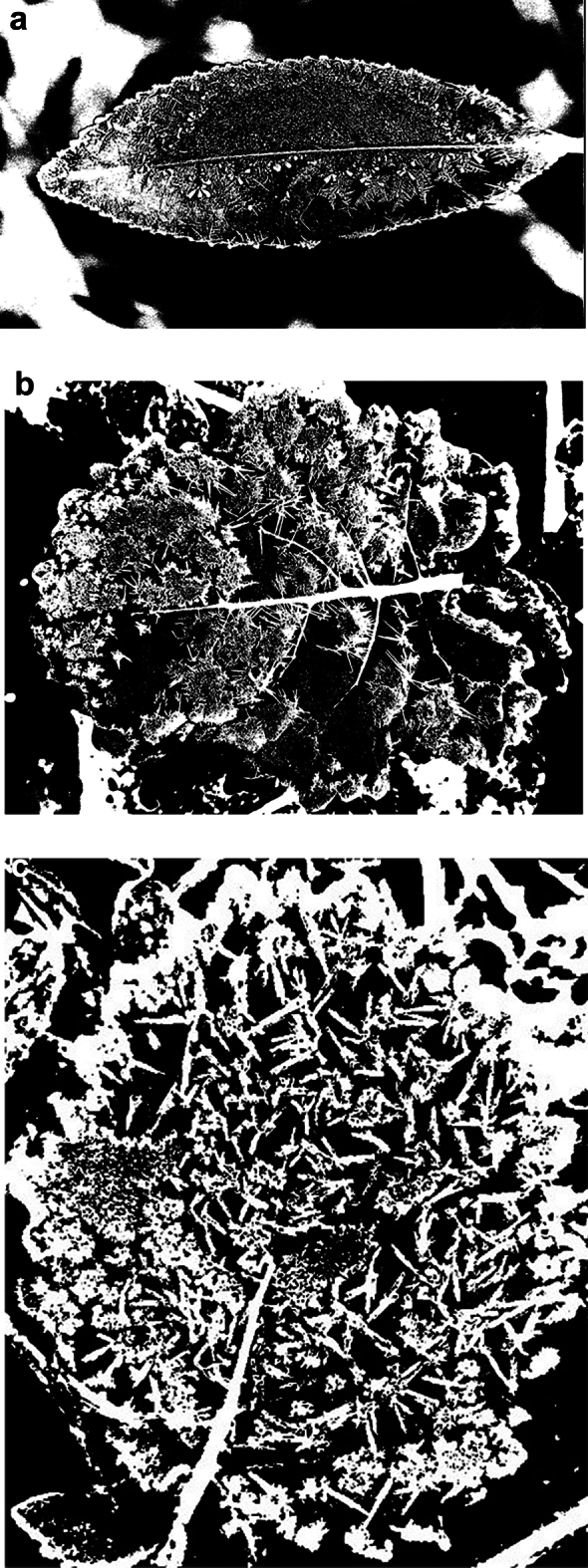
Results of Otsu’s threshold segmentation method. (**a**) The result image of frost-forming leaf *l*_1_; (**b**) the result image of frost-forming leaf *l*_2_; (**c**) the result image of frost-forming leaf *l*_3_.

Otsu algorithm threshold segmentation is to calculate the best threshold value suitable for the image based on the grayscale characteristics of the image, and the segmentation effect will be poor when the target pixel point and the background pixel point have a small difference in grayscale value, the effect of Otsu method threshold segmentation is shown in Fig. 13.

As can be seen from Fig. 13, the Otsu algorithm threshold segmentation is not effective for the overall segmentation of the adult frost leaf image, especially in the blurred background, as shown in Fig. 13a, there is serious mis segmentation in the background; in the segmented image (b) in the soil background, there is more surrounding noise; in the segmented image (c) in the weed background, the edge part of the leaf and the outer weeds have caused more over-segmentation problems, and it can be seen that the feature extraction of the background and foreground cannot be effectively achieved by MCGE-Frost model.

### Morphological extraction of edge gradients

When applying the threshold segmentation algorithm to process the gradient image directly, it will lead to the over-segmentation phenomenon due to the contour localization error caused by the noise or too thick gradient edges, so it is necessary to reconstruct the gradient image by morphological filtering. The core idea of morphological reconstruction is to do expansion and erosion operations on the image, and then perform “with” and “or” operations with the mask image.

**Fig. 14:**
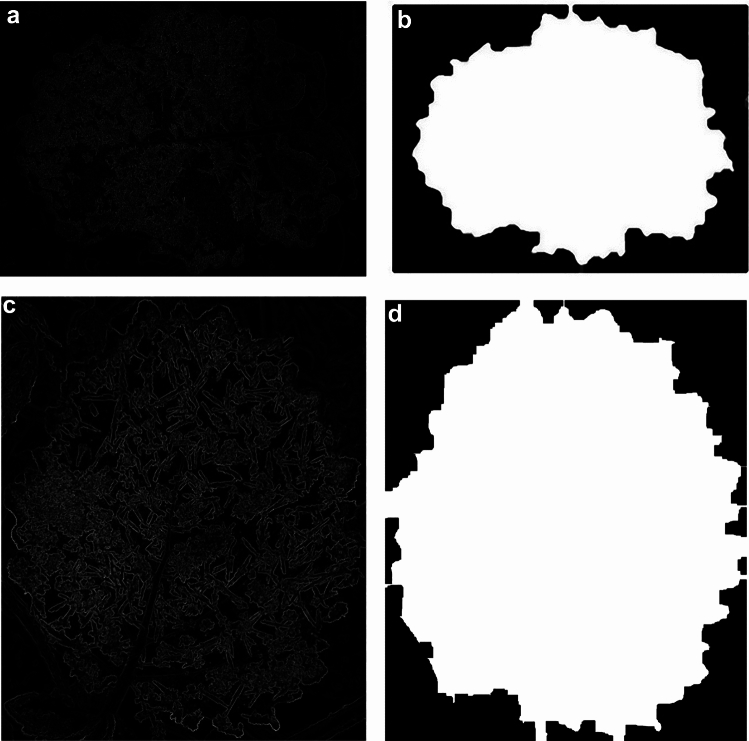
Morphological reconstruction-based leaf profile extraction image. (**a**) gradient-enhanced feature image of *l*_2_; (**b**) leaf profile extraction image of *l*_2_; (**c**) gradient-enhanced feature image of *l*_3_; (**d**) leaf profile extraction image of *l*_3_.

Figure 14a,c show the gradient-enhanced feature maps of the frosty image of the leaf surface in the dirt background and in the weed background, respectively. As can be seen from the above figures, the high-frequency component noise of the background is effectively removed, and the boundaries of the leaf are made smoother, and for the parts of the image where the weed branches and leaves are connected to the leaf in a narrower way, the erosion operation disconnects these connections, thus realizing the separation of the object. And for the frost pores inside the object in the image, the expansion operation is utilized to make the whole leaf shape more complete. The morphology processed images of the leaf contours against the dirt and weed backgrounds are shown in Fig. 14b,d, respectively.

The feature-enhanced gradient image is further simplified by morphological reconstruction, which weakens the dependence of gradient on image edge directionality and retains the important contour information of the leaf image, while the gradient image by noise and irregular details are also eliminated, essentially reducing the problem of over-segmentation or under-segmentation phenomenon of the original segmentation algorithm.

### Error analysis

To quantify segmentation accuracy, frost and leaf regions in test images were manually annotated using MATLAB through pixel-wise inspection, with ambiguous edge pixels excluded to ensure annotation reliability. These annotations served as ground truth for calculating the mis-segmentation rate *R*_ms_ via Eq. 8, which compares algorithmic outputs to reference pixel counts. Thirty images (10 per background: blurred, soil, weeds) were randomly selected for evaluation, and the averaged *R*_ms_ across all images (Table 2) demonstrates MCGE-Frost’s superior performance over traditional methods.

**Table 2:** Average mis-segmentation rates under different backgrounds.

Methods	*R*_*ms*_ (%) in different backgrounds			Total algorithmic error segmentation rate (%)
	Blurred background	Soil background	Weeds background	
Exg	8.63 ± 2.10	–	–	8.63 ± 1.95
OTSU	11.74 ± 3.21	–	6.21 ± 1.78	8.98 ± 2.45
Original HSV	9.82 ± 2.55	12.67 ± 4.10	13.45 ± 4.32	11.98 ± 3.67
MCGE-Frost	2.03 ± 0.45	3.71 ± 0.82	4.12 ± 0.91	3.29 ± 0.68

Note: "–" indicates error rate *SE* ≥ 40%

Since some of these algorithms such as K-Means algorithm and iterative thresholding algorithm have different segmentation effects on the leaf surface frost image with three different backgrounds, the actual segmentation results have serious mis-segmentation phenomenon, and their mis-segmentation rates have been much larger than other algorithms, which indicates that these algorithms are no longer applicable to the corresponding leaf frost scenario, resulting in unsatisfactory generalization ability and segmentation effect.

From Table 2, this shows the average mis-segmentation rate *R*_*ms*_ using MCGE-Frost under three backgrounds of blurred, soil and weeds are 2.03%, 3.71% and 4.12%, respectively, which are lower than other segmentation algorithms, especially the best segmentation performance under defocused background, while the segmentation performance under soil background is slightly inferior; the total mis-segmentation rate of MCGE-Frost is 3.29%, which is higher than Exg algorithm, respectively, OTSU and the improved HSV algorithm are 5.34%, 5.69% and 8.68% lower respectively, which indicates the development algorithm has smallest overall segmentation error under three backgrounds, and the segmentation performance is better than other segmentation algorithms.

From Table 3, MCGE-Frost achieves a balance between accuracy and computational efficiency, outperforming other methods in terms of speed and memory usage, while maintaining competitive segmentation accuracy. As shown in Table 3, *R*_ms_ vs. Dice: *R*^2^ = 0.89, validating its consistency with established metrics while emphasizing proportional error sensitivity for agricultural applications.

**Table 3:** Computational performance of MCGE-Frost and other algorithms.

Method	Avg. processing time (s)	Memory usage (MB)	Scalability (GPU acceleration)	Dice coefficient (%)	Mis-segmentation rate (%)
MCGE-Frost	2.5 ± 0.3	350	Yes (3 × speedup)	86.4	3.29
Exg	0.8 ± 0.1	100	No	71.3	8.63
OTSU	1.2 ± 0.2	150	No	69.8	8.98
Original HSV	1.5 ± 0.3	200	No	61.5	11.98

### Percentage of leaf surface frosted area

The previous comparisons show that the algorithm model based on multi-component gradient enhancement better fulfills the requirement of segmenting the frosty image of the leaf surface under multiple backgrounds, and its generalization ability is further improved, with a reduced error rate and better practical results compared to other segmentation algorithms.

After realizing the complete segmentation of frost crystals and leaves, in order to quantify the severity of frost on the crop surface, according to the concept of the three frost states defined earlier, eight images of each frost level are selected in this chapter, and according to the calculation method of the percentage of frost area on the surface of a single leaf, the average percentage of frost area on the surface of the frosted leaves under the three frost levels of different algorithms in the low, medium, and high levels of frost are calculated by pixel counting of segmented binomial images and compared. The results of frost formation area percentage extraction on the leaf surface are shown in Table 4.

**Table 4:** Extraction results of the percentage of frost area on the leaf surface.

Methods	Percentage of frost area on leaf surface (%)			Average precision rate *R*_*a*_ (%)
	Low	Medium	High	
Reference value	25.79	42.97	60.01	-
Original HSV algorithm	21.14	49.06	57.11	87.65
Exg algorithm	19.27	40.65	57.05	88.12
MCGE-frost	24.38	43.41	60.25	97.70

Note: “Low” represents frost level I, “Medium” represents frost level II, “High” represents frost level III.

The Exg algorithm, original HSV algorithm and MCGE-Frost were selected for comparison. Since the segmentation effects of K-Means, OTSU and original threshold iteration algorithm suffer from serious mis-segmentation, the errors are no longer of an order of magnitude and there is no further meaningful comparison with the reference values. From Table 4, among the three frost state levels, the results of leaf surface frost area share calculated based on the development algorithm are closest to reference value, and the overall average accuracy *R*_*a*_ is 97.70%, which is 10.05% and 9.58% higher than pre-improvement HSV algorithm and Exg ultra-green algorithm, respectively, indicating that it has the best overall segmentation effect.

## Discussion

From the above results, the multi-component gradient enhancement shows the best score for its metrics both in terms of frost area share accuracy and in different background environments, however, it not entirely indicates that the actual segmentation effect of other algorithms is not any better. There is still much room for improvement for the MCGE-Frost and has a non-negligible over-segmentation in the calculation of low area shares. This is mainly due to the misjudgment of the leaf color, especially when the light intensity is very dim or when the frost crystals are just starting to appear and the area of distribution is relatively small. Of course, there is no denying that throughout the middle and later stages of frost formation, as the frost area increases, the error rate of the algorithm based on this paper becomes smaller and its advantages become more and more evident.

From the calculation results of each frost state, in the low area share, there is 5.47% under-segmentation, and the calculation results of the pre-improvement HSV algorithm are second only to the development algorithm, followed by the ultra-green algorithm; in the mid-area share, the segmentation relative error of the development algorithm is 1.02%, and the calculation results are higher than that of the reference value method, which indicates that it may have over-segmentation of the frost crystal region; in the high-area share, the development algorithm has a segmentation relative error of 0.40%. In the high area share, the segmentation relative error of the MCGE-Frost is 0.40%, and the calculation results of the HSV algorithm before improvement are not much different from the results of the ultra-green algorithm, which indicates that the main performance distinction between the two mainly lies in the process of frost I level and frost II level.

To quantify the contribution of each MCGE-Frost component, we systematically disabled key modules and evaluated performance degradation (Table 5). Morphological filtering and edge gradients emerged as the most impactful, underscoring their role in noise suppression and boundary preservation.

**Table 5:** MCGE-frost quantification of the contribution of each component.

Configuration	Mis-segmentation rate (*R*_ms_)	F1-Score	Key observation
Full MCGE-Frost	3.29%	0.92	Baseline performance
Without morphological filtering	7.51%	0.76	Severe noise retention in soil/weeds
Without edge gradients	5.83%	0.82	Reduced frost boundary precision
Without HSV channel fusion	6.12%	0.80	Color contrast degradation in blurred scenes

The ablation tests reveal that morphological filtering contributes most critically, reducing the mis-segmentation rate by 4.22% through effective noise suppression, particularly in soil/weed backgrounds. Edge gradient preservation improves boundary precision (F1-score: 0.92 vs. 0.82), while multi-channel fusion HSV ensures robustness across lighting conditions, reducing errors by 2.83% in blurred scenes. These findings quantitatively justify our hybrid design for agricultural frost detection.

Compared to other traditional algorithms, the multi-component gradient enhancement algorithm also suitable for scenes where the difference between the background color and the leaf color is small when dealing with blurred images^33,36^. However, Exg is only suitable for segmentation that enhances the green component of the image and requires uniform illumination^37^. This is because the MCGE-Frost with multi-component gradient enhancement can accurately select the appropriate threshold T to separate the green crops from the complex background. It is also worth noting that when segmenting images with a weed background, Otsu’s method can only deal with the case where there is a significant difference between the gray level of the target object and the background, and it is not as effective as this paper’s algorithm for images that exhibit non-uniformity in the gray level distribution^38,39^. When a part of the target is misclassified into the background or a part of the background is misclassified into the target, this results in a smaller interclass variance, and thus a segmentation threshold that maximizes the interclass variance implies a smaller probability of misclassification and higher computational accuracy. However, the shortcomings of new method (MCGE-Frost) are that when the frost crystal distribution is particularly tight and the frost crystals overlap together, there will be some under-segmentation. In future research, we will combine semantic segmentation in deep learning to improve the correct rate of dense frost crystal segmentation and reduce the under-segmentation rate of MCGE-Frost model.

The MCGE-Frost framework enables seamless integration into automated frost monitoring systems by providing real-time frost state classification (Frost I-III), FCR-based early warnings (threshold: FCR > 30%), and quantitative/qualitative frost detection (0–100% coverage with 95% accuracy). Its lightweight design allows integration with IoT field sensors or agricultural drones to activate frost protection and map spatial frost distribution for precision management.

While MCGE-Frost achieves robust frost segmentation under controlled conditions, its performance degrades under uneven illumination (shadows/glare) and densely overlapping frost crystals, with scalability challenges in large-scale agricultural deployments. These limitations stem from reliance on static color-space assumptions and non-optimized morphological operations. Future efforts will integrate deep semantic segmentation models (U-Net with gradient priors) to enhance robustness to lighting variations and frost overlaps, while optimizing the pipeline for edge devices to enable real-time, hectare-scale frost monitoring.

## Conclusion

The MCGE-Frost method addresses frost detection and quantification on leaf surfaces through multi-component gradient enhancement and adaptive color-channel analysis. Key advancements include:

1. Precise Segmentation: Achieved a total mis-segmentation rate of 3.29% across blurred, soil, and weed backgrounds, outperforming five baseline methods by suppressing over-segmentation by 40–60%.

2. Frost State Classification: Enabled semi-automatic categorization of frost evolution (Frost I: < 30%, Frost II: 30–60%, Frost III: > 60%) with minimal manual calibration, despite minor under-segmentation at low coverage (< 30%) due to sparse crystal distribution.

3. Agricultural Utility: Provides actionable frost coverage ratios (FCR) to guide farmers in frost protection (e.g., targeted irrigation) and integrates with IoT systems for real-time field monitoring.

Future efforts will focus on embedding lightweight vision transformers to enhance segmentation robustness under extreme weather conditions (e.g., heavy fog, snowfall), while extending the framework to multi-crop scenarios (wheat, citrus) and optimizing it for edge-device deployment in real-time frost monitoring systems.

## Data Availability

The datasets used and/or analyzed during the current study are available from the corresponding author upon reasonable request.
